# Toxicity assessment of *Cucurbita pepo cv Dayangua* and its effects on gut microbiota in mice

**DOI:** 10.1186/s12906-024-04551-w

**Published:** 2024-06-22

**Authors:** Huan Zhang, Yazhou Zhou, Zhiyuan Pan, Bikun Wang, Lei Yang, Nan Zhang, Baiyi Chen, Xiaona Wang, Zhiguang Jian, Likun Wang, Hui Ling, Xiaoming Qin, Zhelin Zhang, Teng Liu, Aiping Zheng, Yafang Tan, Yujing Bi, Ruifu Yang

**Affiliations:** 1 School of Public Health, Hebei Medical University, Shijiazhuang, 050017 China; 2 State Key Laboratory of Pathogen and Biosecurity, Academy of Military Medical Sciences, Beijing, 100071 China; 3 State Key Laboratory of Toxicology and Medical Countermeasures, Institute of Pharmacology and Toxicology, Beijing, 100850 China; 4 Heilongjiang Biodi Bio-Pharma Technology Company Lmt., No. 178, Yuexiujie, Harbin, Heilongjiang Province China

**Keywords:** *Cucurbita pepo cv Dayangua*, Acute toxicity, Sub-chronic toxicity, Gut microbiota

## Abstract

**Background:**

*Cucurbita pepo cv Dayangua* (CPD) is an edible plant with diverse pharmacological properties. The current research on CPD has primarily focused on initial investigations of its chemical composition and pharmacological effects, and no comprehensive toxicity assessment has been conducted to date.

**Methods:**

In the present study, the toxicity of CPD was evaluated through both acute and sub-chronic oral toxicity tests in mice. 16S rDNA sequencing was used to analyze the composition of the gut microbiota of mice at different time points to observe the effect of CPD on these microbial communities.

**Results:**

In the acute toxicity test, CPD exhibited low toxicity, with a median lethal dose (LD50) > 2000 mg/kg. The sub-chronic toxicity test indicated that CPD administration at doses of 200, 400, and 600 mg/kg did not cause mortality or significant organ damage in mice. Furthermore, analysis of the gut microbiota after gavage administration of CPD at 400 and 600 mg/kg revealed an improved abundance of some beneficial gut bacteria.

**Conclusions:**

In summary, no acute or sub-chronic toxic effects were observed in mice following the oral administration of CPD. CPD did not affect the structure and diversity of the gut microbiota and may contribute to an increase in the number of beneficial gut bacteria.

## Introduction

Various recent studies have confirmed the pivotal role of functional foods and herbal medicines in disease treatment [1]. Moreover, many natural edible plants have been receiving increased attention for health promotion and disease prevention because of their richness in beneficial components and low side effect profiles [2]. As one of the most important families of natural food plants, the *Cucurbitaceae* consists of approximately 100 genera. Zucchini (*Cucurbita pepo Linn*) is a representative edible plant in the *Cucurbitaceae* family and is cultivated globally as a crop with economic and nutritional value [3]. Zucchini is rich in vitamins C and A, carotenoids, potassium, and phenolic compounds [4], and is known to possess anti-inflammatory and antioxidant properties [5].

*Cucurbita pepo cv Dayangua* (CPD) is a variety of zucchini primarily found in Duolun County, Inner Mongolia [6]. CPD, locally known as “Dakugua”, has a bitter taste and beyond its status as an edible plant it is also valued as a local herbal medicine to treat common colds, with effects such as relieving fatigue, analgesia, and anti-diarrhea [7]. CPD contains a variety of cucurbitacins, including cucurbitacin B, 23,24-dihydro cucurbitacin D, and cucurbitacin E [8]. Other studies have isolated chemical CPD components including β-sitosterol and succinic acid [9, 10]. These ingredients have been shown to inhibit tumor cell proliferation [11] and exert antioxidant [12, 13] and anti-inflammatory activities [14]. Several studies have investigated the pharmacological effects of CPD. One study indicated that the crude CPD extract exerts anti-inflammatory effects on paw edema and cotton pellet-induced granuloma in rats [15]. Other studies have shown that CPD fruit extract exhibits analgesic activity against sharp pain caused by thermal stimulation [16] and inhibits the proliferation of canine parvovirus and *Staphylococcus aureus* [17]. These results suggest that CPD has a wide range of pharmacological effects and may be valuable for therapeutic applications. In some countries, plants with therapeutic properties, such as ginseng, wolfberry, and lotus seeds, are routinely used as functional foods and dietary supplements, reflecting the concept of homology between medicine and food [18, 19]. Therefore, CPD may have a similar potential for use as a functional food.

Although numerous natural plants can exert many beneficial functions [20, 21], they can also have toxic side effects during use, which may be consequential for human health [22]. The current research on CPD is mostly limited to preliminary studies on its chemical composition and pharmacological effects, and no systematic evaluation of CPD toxicity has been conducted to date. Therefore, to facilitate the therapeutic application of this plant and ensure safety, a comprehensive understanding of its toxicity profile is pertinent.

With the development of microbiome studies in recent years, various findings have revealed the composition and function of the gut microbiota [23]. The gut microbiota is considered to be a vital “organ” that exerts a significant influence on the physical and mental health of individuals by performing essential functions in the metabolic, immune, structural, and nervous systems of the body [24]. Interactions exist between diet, gut microbiota, and the host [25]. Food is considered a key factor that shapes the composition of the gut microbiota and the gut environment [26]. The gut microbiota plays an important role in the maintenance of drug-host interactions and mediates the treatment process of many Chinese herbal medicines [27]. Studies have demonstrated an interaction between ginseng and gut microbiota. Ginseng modulates the composition of the gut microbiota, which is involved in ginseng metabolism in the host [28]. Various types of tea can also regulate the gut microbiota and exert beneficial effects [29]. Gut microbiota has also been associated with the alleviation of host obesity by fermented tomatoes [30]. As an edible plant, CPD has been shown to have pharmacological effects. CPD may also have a regulatory effect on the gut microbiota. However, to the best of our knowledge, no studies have reported the beneficial or adverse effects of CPD on the gut microbiota. Therefore, delineating the effect of CPD on gut microbiota could provide more information for its application.

The purpose of this study was to systematically evaluate the toxicity of CPD through both evaluation of acute and sub-chronic oral toxicity in mice and to explore its potential effects on the gut microbiota to provide evidence for further research on the pharmacological effects of CPD and the development of nutraceutical products in the future.

## Materials and methods

### Preparation of plant material

CPD fruits were provided by the Heilongjiang Biodi Bio-Pharma Technology Company, Ltd. (Heilongjiang Province, China) and identified by Prof. Guiming Liu of the Institute of Biotechnology, Beijing Academy of Agriculture and Forestry Sciences. Voucher specimens (IB2023-010) were deposited at the Institute of Biotechnology, Beijing Academy of Agriculture and Forestry Sciences. The plant material was used with the permission of Heilongjiang Biodi Bio-Pharma Technology Company, Ltd. Clean and dried fruits were ground into a powder and then filtered through a 250-mesh sieve. After filtration the particle size of the powder was determined using a laser particle sizer (Sympatec, HELOS-RODOS), the mean particle size of CPD ranges from 20–30 µm. The sieved powder was weighed and prepared into a suspension with distilled water at a concentration of 100 mg/mL, which was subsequently homogenized using a homogenizer (FLUKO, FM200A) for 3 min to uniformly disperse the suspension to enable the prepared CPD suspension to be smoothly passed through the gavage needle of mice. The final CPD solution was prepared with distilled water at the specified dose concentration and stored at -80 °C until use.

### Experimental animals

Specific-pathogen-free male and female C57BL/6 mice (6–8 weeks old, weighing 18 − 22 g) were obtained from Beijing Vital River Laboratory Animal Technology Co., Ltd. (Beijing, China). The animals were kept in a controlled environment at a temperature of 23 ± 2 °C and relative humidity of 40 ± 5%, with food and water ad libitum, and with a 12-h light and dark cycle. All animal experimental protocols were approved by the Animal Ethics Committee of the Animal Laboratory Center in Academy of Military Medical Sciences (IACUC-DWZX-2021013). All animals were acclimatized for one week before the start of the experiment.

### Acute oral toxicity study

The exploration of the acute oral toxicity of CPD was conducted in accordance with the “Up-and-Down-Procedure (UDP)” described in the Organization for Economic Cooperation and Development (OECD) guideline 425 [31]. CPD, an edible plant, is presumed to have low toxicity. Therefore, a limit test was performed at a dose of 2000 mg/kg. A total of 20 animals were randomly divided into 4 groups (*n* = 5): male CPD group (M-2000 mg/kg), male control group (M-Control), female CPD group (F-2000 mg/kg), and female control group (F-Control). Initially, after a 4-h of fasting but not water, a mouse from the M-2000 mg/kg group was administered 2000 mg/kg CPD by oral gavage, while a mouse from the M-Control group was administered the same volume of distilled water. If the first mouse survived, four additional animals were administered the same dose sequentially so that a total of five animals were tested; female mice were subjected to the same procedure. The animals were given special attention for the first 4 h after administration, followed by daily observations of behavior and mortality until they were humanely executed after 14 days.

Mortality, food intake, and body weight of mice were recorded during the experiment. Important parameters such as diarrhea, respiration, fur shrugging, and mental status were also observed. After the experiment, the mice were anesthetized using an injection of sodium pentobarbital (40 mg/kg), and blood was collected from the retro-orbital venous plexus. Subsequently, the mice were euthanized by gradually increasing concentrations of carbon dioxide at a flow rate of 5 L/min. Finally, the vital organs of the mice were harvested for subsequent analyses.

### Sub-chronic oral toxicity study

Male mice were randomly assigned to 4 groups (*n* = 7–8), including a control group and 3 CPD test groups. Based on the dose design of the acute toxicity test, the intervention dose of CPD was set at approximately 1/10, 1/5, and 1/3 of 2000 mg/kg in the sub-chronic test. The mice in the CPD groups were administered daily doses of 200, 400, or 600 mg/kg CPD by oral gavage for 12 weeks. The control group was administered an equivalent volume of distilled water daily. The animals in each group were monitored daily for their general status and toxicity symptoms, and their food intake was recorded and weighed weekly. To analyze the changes in the gut microbiota of the mice in each group, fresh fecal samples were collected and stored at -80 °C until use.

At the end of the experiment, the mice were anesthetized using an injection of sodium pentobarbital (40 mg/kg), and blood was collected from the retro-orbital venous plexus. Subsequently, the mice were euthanized by gradually increasing concentrations of carbon dioxide at a flow rate of 5 L/min. Vital organs were harvested after the mice were euthanized to observe any noticeable lesions. Relative organ weight was calculated as follows [32]:

$$\mathrm{Relative}\;\mathrm{organ}\;\mathrm{weight}\;=\;\mathrm{weight}\;\mathrm{of}\;\mathrm{an}\;\mathrm{organ}\;(\mathrm g)\;/\;\mathrm{body}\;\mathrm{weight}\;\mathrm{of}\;\mathrm{the}\;\mathrm{mice}\;\mathrm{on}\;\mathrm{the}\;\mathrm{day}\;\mathrm{of}\;\mathrm{sacrifice}\;(\mathrm g)\times100$$ 

### Hematological and serum biochemical analysis

Blood samples collected in anticoagulant-containing tubes in the sub-chronic toxicity test were used to determine hematological parameters, including white blood cell count (WBC), red blood cell count (RBC), lymphocyte count (Lymph#), monocyte count (Mon#), granulocyte count (Gran#), hemoglobin (HGB), hematocrit (HCT), platelet count (PLT), red blood cell distribution width (RDW) and mean corpuscular hemoglobin concentration (MCHC). These hematological parameters were determined using an auto hematology analyzer (Mindray, BC-2800vet).

Anticoagulant-free blood samples from mice in the acute and sub-chronic oral toxicity tests were used for serum biochemical assays. Biochemical parameters in the acute toxicity test included alanine aminotransferase (ALT), aspartate aminotransferase (AST), serum urea (UREA), creatine (CREA), cholesterol (CHO), triglycerides (TG), low- density lipoprotein (LDL), and high-density lipoprotein (HDL) levels. In addition to the above biochemical parameters, we determined the levels of serum lactate dehydrogenase (LDH) and creatine kinase (CK) in the sub-chronic toxicity study to better assess the toxicity of CPD. Biochemical analysis was performed using an automatic biochemical analyzer (Rayto, Chemray 800).

### Histological analysis

The hearts, livers, spleens, lungs, kidneys, and colons of the mice were collected and fixed in a 4% tissue fixative solution. After paraffin embedding, the tissues were sectioned with a microtome (Leica, RM2016), stained with hematoxylin and eosin (H&E), and analyzed subsequently.

### Gut microbiota analysis

The gut microbiota of mice in the sub-chronic toxicity was analyzed by 16S rDNA gene amplicon sequencing, which was performed by Novogene Biological Technology Company (Beijing, China). Total genomic DNA was extracted from the mouse fecal samples using the CTAB method. The 16S rDNA genes in distinct regions (16S V3-V4) were amplified with specific primers (F: CCTAYGGGRBGCASCAG; R: GGACTACNNGGGTATCTAAT) and barcodes. For the analysis of the results, the indices of alpha diversity were used to reflect the richness and uniformity of the communities in the sample, whereas beta diversity was calculated to evaluate the complexity of the community composition and compare the differences between groups. The Linear discriminant analysis effect size (Lefse) analysis was performed to identify biomarkers with statistical differences between groups [33]. The raw data were uploaded to the NCBI SRA database (BioProject ID: PRJNA976252).

### Statistical analysis

Statistical analyses were carried out using GraphPad Prism software (version 9.0.0). The data were expressed as means ± standard deviation (SD), median and interquartile range (IQR), or as box plots ranging from minimum to maximum values when appropriate. Statistical significance was evaluated through one-way ANOVA followed by Tukey’s test for multiple comparisons or the non-parametric Kruskal–Wallis test with Dunn's multiple comparison test. Statistical significance was set at *P* < 0.05.

## Results

### Acute oral toxicity study

#### Mortality and LD50

CPD did not cause mortality in male or female mice at a dose of 2000 mg/kg, and no mortality occurred in the control group. Diarrheal symptoms were observed in all CPD-treated mice within the first 24 h, which recovered at 48 h post-administration. No other apparent adverse responses were observed in mice of either sex during the period of 14 days. Thus, based on the limit test, the LD50 of CPD in mice by oral administration was estimated to exceed 2000 mg/kg.

### Effects of CPD on body weight, food intake, and biochemistry parameters of mice in acute oral toxicity study

**Fig. 1:**
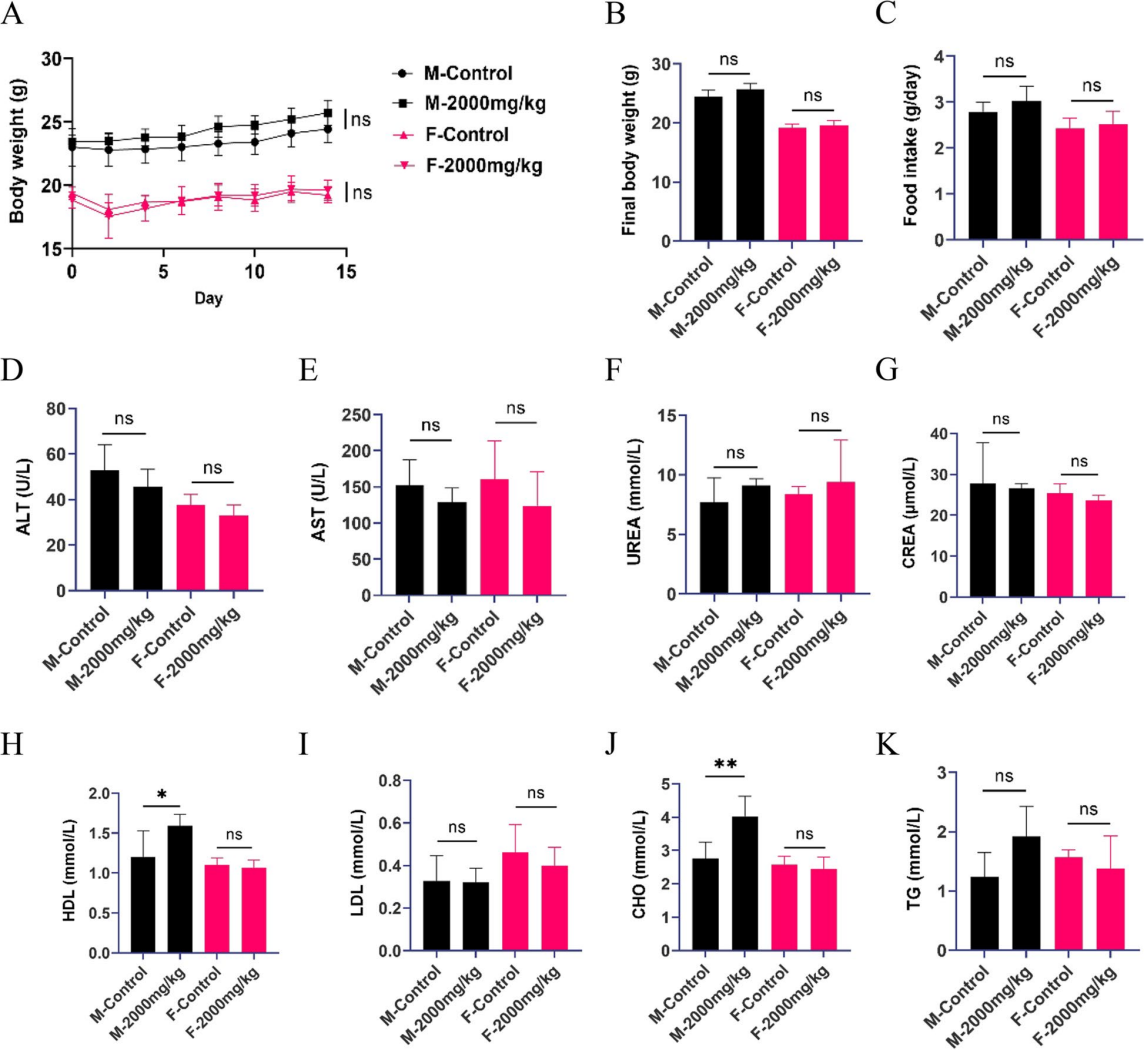
Effects of CPD on body weight, food intake, and serum biochemistry parameters in mice in acute toxicity study for 14 days. F-control or M-control, which female or male mice were treated with water; F-2000 mg/kg or M-2000 mg/kg, which female or male mice were treated with 2000 mg/kg of CPD. **A**-**C** Bodyweight and food intake of mice; **D**-**E** Liver function enzymes (ALT and AST); **F**-**G** The indicators of renal function (UREA and CREA); **H–K** The levels of lipoproteins (HDL, LDL, CHO, and TG); ns, not statistically significant. ^*^ *P* < 0.05, ^**^ *P* < 0.01. Data are expressed as Mean ± SD

CPD did not cause significant differences in body weight or food intake in female or male mice throughout the observation period compared to the control group (Fig. 1A-C). For serum lipoproteins, CPD had no effects on the levels of LDL and TG (Fig. 1I and K). In contrast, CPD significantly increased the levels of HDL and CHO in male mice compared to those in the control group but had no effects on female mice. However, the changes in HDL and CHO levels were fluctuations within the normal ranges (Fig. 1H and J). In addition, there were no significant differences in ALT and AST, which are indicators of liver damage, or in UREA and CREA, which reflect renal function in either female or male mice between control and treated groups (Fig. 1D-E and F-G).

### Pathological lesions in the major organs of mice in acute oral toxicity study

**Fig. 2:**
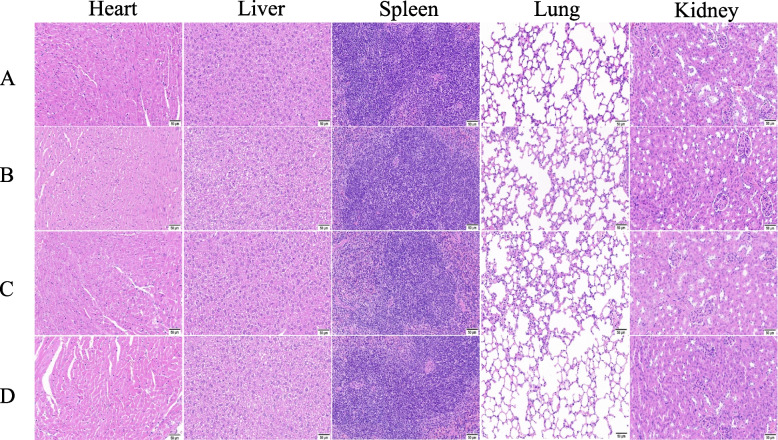
Representative histological images of heart, liver, spleen, lung, and kidney of male and female mice from different groups in the acute toxicity study. **A** Histological images of female mice in the control group; **B** Histological image of female mice treated with 2000 mg/kg CPD; **C** Histological images of male mice in the control group; **D** Histological image of male mice treated with 2000 mg/kg CPD; Scale bar: 50 μm

Besides the observation of the mortality and general status, we examined whether there were any pathological changes in the major organs of the mice. Sections of the heart, liver, spleen, lungs, and kidneys of the mice in all groups showed normal histomorphology (Fig. 2). Histological images of the mice in the different groups showed the structural integrity of the organs without significant necrosis or inflammatory cell infiltration.

### Sub-chronic oral toxicity study

#### Effects of CPD on body weight, food intake, and biochemistry parameters of mice

**Fig. 3:**
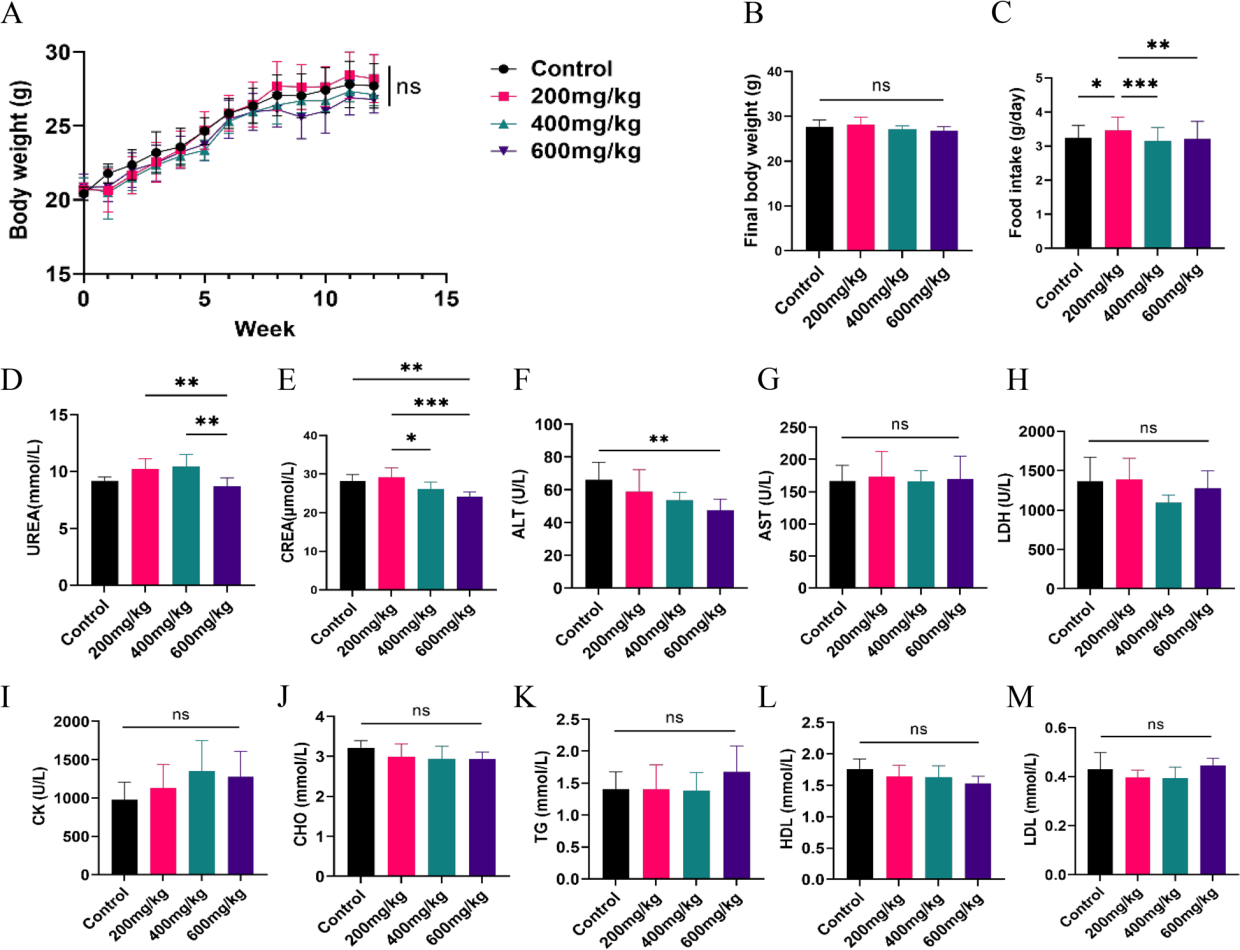
Effects of different doses of CPD on body weight, food intake, and serum biochemistry parameters of male mice in sub-chronic toxicity study. **A**-**C** Body weight and food intake of mice. **D**-**E** The indicators of renal function (UREA and CREA); **F-G** Liver function enzymes (ALT and AST); **H-I** Myocardial enzymes (LDH and CK); **J-M** Lipoproteins (CHO, TG, HDL, and LDL). ns, not statistically significant, ^*^ *P* < 0.05, ^**^ *P* < 0.01 and ^***^ *P* < 0.001. Data are expressed as Mean ± SD

The changes in the body weights of the mice were monitored over 12 weeks, and there were no significant differences between the groups (Fig. 3A-B). In comparison with the control group, the administration of CPD did not reduce the food intake of the mice, and a dose of 200 mg/kg increased food consumption (Fig. 3C). To investigate the sub-chronic toxicity of CPD in mice, we examined alterations in markers of liver function (ALT and AST) and kidney function (UREA and CREA) as well as changes in cardiac enzymes and serum lipoproteins. The results showed that CPD did not affect the levels of the cardiac enzymes LDH and CK or the levels of the lipoproteins CHO, TG, HDL, and LDL when compared with those in the control group (Fig. 3H-M). CPD had no effect on the concentrations of UREA and AST, but a dose of 600 mg/kg significantly reduced the concentrations of CREA and ALT compared with those in the control group (Fig. 3D-G). However, the reduced levels of CREA and ALT remained within the normal reference values. These results imply that CPD intervention caused no noticeable myocardial, hepatic, or renal damage after 12 weeks, nor did it disturb normal serum lipoprotein levels.

### Relative organ weights

To observe whether the organ weights of the mice changed at the end of the experiment, we calculated the relative weights of important organs such as the heart, liver, spleen, lungs, kidneys, and testes, and the results are shown in Table 1. No significant differences were found in the relative weights of the organs among the groups. Long-term supplementation with different CPD doses elicited no changes in organ weights in mice, implying its low toxicity.

**Table 1:** The relative organ weights of male mice in sub-chronic toxicity study

Relative organ weight (%)				
	Control	200 mg/kg of CPD	400 mg/kg of CPD	600 mg/kg of CPD
Heart	0.69 ± 0.05	0.71 ± 0.07	0.64 ± 0.12	0.62 ± 0.03
Liver	4.90 (0.30)	5.32 ± 0.46	5.21 ± 0.22	5.05 ± 0.39
Spleen	0.25 ± 0.03	0.25 (0.08)	0.24 ± 0.03	0.26 (0.04)
Lung	0.65 ± 0.08	0.62 ± 0.06	0.63 ± 0.04	0.57 ± 0.09
Kidney	1.21 ± 0.05	1.21 (0.12)	1.21 ± 0.07	1.19 ± 0.04
Testes	0.62 ± 0.05	0.66 ± 0.08	0.64 ± 0.12	0.62 ± 0.10

Data are expressed as Mean ± SD or Median (IQR)

### Hematological parameters

Hematological parameters are shown in Table 2. No remarkable differences were observed in any of the hematological parameters measured between the groups. The results revealed that the supplementation of CPD for 12 weeks did not affect the hematological parameters of mice.

**Table 2:** Hematological parameters of male mice in sub-chronic toxicity study

	Control	200 mg/kg of CPD	400 mg/kg of CPD	600 mg/kg of CPD
WBC (10^9^/L)	7.70 ± 1.33	8.51 ± 1.93	7.78 ± 1.53	9.69 ± 1.70
RBC (10^12^/L)	10.99 ± 0.44	10.98 ± 0.63	10.96 ± 0.49	11.14 ± 0.73
HGB (g/L)	159.00 ± 8.07	156.30 ± 9.32	156.40 ± 7.27	158.10 ± 11.77
PLT (10^9^/L)	1934.00 ± 218.90	2067.00 ± 271.20	1778.00 ± 165.50	2000.00 ± 218.10
HCT (%)	56.96 ± 2.67	56.54 ± 2.46	57.90 (5.00)	55.67 ± 3.24
Lymph# (10^9^/L)	6.08 ± 1.00	6.85 ± 1.69	6.16 ± 1.20	7.96 ± 1.29
Mon# (10^9^/L)	0.19 ± 0.06	0.20 ± 0.05	0.16 ± 0.07	0.20 ± 0.06
Gran# (10^9^/L)	1.44 ± 0.46	1.46 ± 0.27	1.35 (0.38)	1.53 ± 0.38
MCHC (g/L)	278.60 ± 7.43	275.90 ± 6.60	277.50 (8.00)	283.30 ± 6.26
RDW (%)	16.33 ± 0.57	16.11 ± 0.73	16.30 (0.55)	16.30 (0.10)

Data are expressed as Mean ± SD or Median (IQR)

### Histopathological analysis

**Fig. 4:**
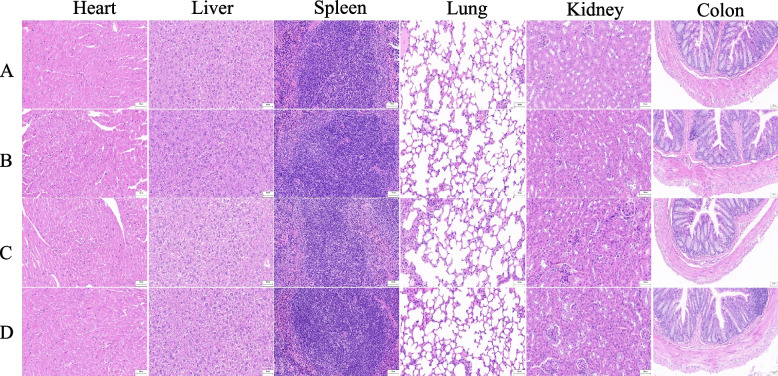
Histopathological analysis of heart, liver, spleen, lung, kidney, and colon in male mice after oral administration of CPD in sub-chronic toxicity study. **A** Representative images of tissues from control mice; **B** Representative images of tissues from mice treated with 200 mg/kg CPD per day; **C** Representative images of tissues from mice treated with 400 mg/kg CPD per day; **D** Representative images of tissues from mice treated with 600 mg/kg CPD per day; Scale bar:50 μm

Pathological changes in the vital organs of the mice were examined. As shown in Fig. 4, H&E staining demonstrated normal morphology and structural features of the heart, liver, spleen, lung, kidney, and colon of both control and CPD-treated mice. The organs were not significantly damaged and no infiltration of inflammatory cells was observed. Exposure to the highest dose (600 mg/kg) for 12 weeks caused no noticeable pathological changes in the organs of the mice, suggesting the safety of CPD.

### Effects of CPD on gut microbiota

**Fig. 5:**
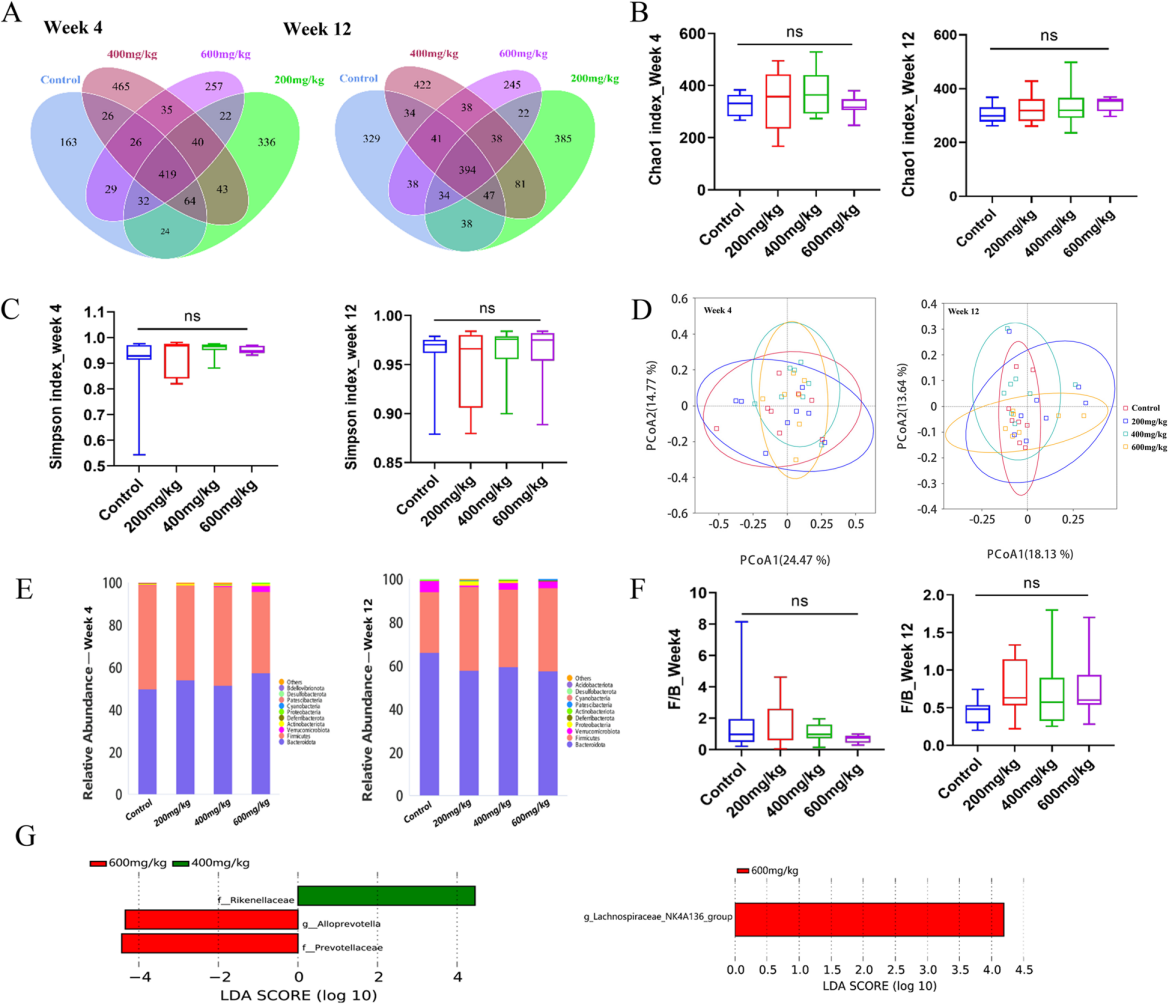
Effects of CPD on the gut microbiota of male mice in week 4 and week 12. **A** Venn diagram showing unique and common ASVs across groups; **B**-**C** The Chao1 and Simpson indices reflecting alpha diversity of the gut microbiota; **D** Principal coordinate analysis (PCoA) of gut microbiota based on the Bray_Curtis distance; **E** Community bars for the top 10 abundances at the phylum level for the four groups at different sampling times, with *Firmicutes* and *Bacteroidetes* being the most abundant species in all samples; **F** The ratio of abundance of *Firmicutes* and *Bacteroidetes*; **G** Lefse analysis showed significantly different bacterial taxa among different groups with LDA score > 4

As an important contributor, diet can exert a considerable influence on the composition as well as the function of the gut microbiota [34]. To observe the effects of CPD on the gut microbiota of mice, we analyzed the composition and changes in the gut microbiota at weeks 4 and 12 by performing 16S rDNA gene sequencing. The Venn diagram shows that the four groups shared 419 and 394 Amplicon Sequence Variants (ASVs) at weeks 4 and 12, respectively. Unique ASVs were observed among groups (Fig. 5A). The alpha diversity was reflected by the Chao1 and Simpson indices, and the results indicated no significant differences in the richness and evenness of the gut microbiota in mice among the groups at weeks 4 and 12 (Fig. 5B-C). Beta diversity of microbial communities was assessed using Principal Co-ordinates Analysis (PCoA) based on the Bray_Curtis distance, and the results showed no significant separation of gut microbiota among groups at different time points (Fig. 5D). The alpha- and beta-diversity results revealed that CPD intervention did not perturb the community structure or diversity of the gut microbiota in mice.

The relative abundances of the top 10 microbial taxa at the phylum level suggested that the highest relative abundances were found in *Firmicutes* and *Bacteroides*, and the ratio of *Firmicutes* to *Bacteroides* (F/B) was not significantly different among the groups at weeks 4 and 12 (Fig. 5E-F). A Lefse analysis was performed to identify statistically different biomarkers among the groups, and the results showed that *Rikenellaceae* was enriched after an intervention of 400 mg/kg CPD for 4 weeks. In contrast, 600 mg/kg significantly increased the abundances of *Alloprevotella* and *Prevotellaceae*. When the intervention was performed for 12 weeks, CPD at 600 mg/kg showed significant enrichment in *Lachnospiraceae_NK4A136_group* (Fig. 5G).

## Discussion

With increasing health consciousness, there is a growing interest in utilizing natural herbal remedies and functional foods with beneficial effects for disease management [35]. Consequently, plant-derived medicinal products are gaining increasing attention. However, it is important to consider the toxic side effects of these substances, because they have important implications for human health. CPD has several pharmacological effects and is traditionally used to treat colds and diarrhea. However, limited research has been conducted on CPD toxicity. In this study, the acute and sub-chronic oral toxicities of CPD were evaluated. In the acute toxicity study, we found that a dose of up to 2000 mg/kg did not cause mortality or pathological changes in the major organs of mice. Food intake, body weight, and serum biochemical parameters showed no adverse changes after the CPD intervention. The LD50 of CPD was estimated to be greater than 2000 mg/kg in our study, according to the Globally Harmonized Chemical Classification and Labeling System (GHS), CPD is classified as category 5 [36, 37], which implies its low toxicity.

Based on acute toxicity, sub-chronic toxicity studies provide a more in-depth understanding of a substance's effects, especially for medicinal plants that require repeated use [32]. In the present study, the sub-chronic toxicity of CPD was evaluated. Mice were administered 200, 400, or 600 mg/kg CPD for 12 weeks, and toxicity changes were observed at the end of the experiment. We found that, in comparison with the control group, the intervention of CPD did not induce a suppressive effect on body weight and appetite in mice (*P* > 0.05), with a dose of 200 mg/kg even promoted food intake (*P* < 0.05), suggesting that CPD does not affect the general growth status of mice. Hematological and serum biochemical parameters are a comprehensive reflection of the degree of damage to an organism after systemic exposure to toxicants. Consistent with other toxicity assessment studies, we examined hematological parameters and serum enzymes that reflect liver and kidney function in mice [38, 39]. In addition, cardiac enzymes and lipoprotein levels in serum were measured. In the acute toxicity study, there were no significant toxic alterations in serum biochemical parameters in mice treated with 2000 mg/kg of CPD. The results of the sub-chronic toxicity study showed that there were no significant changes in hematological parameters in mice compared to the control group (*P* > 0.05). For serum biochemical parameters, the reduction of CREA and ALT was induced only by 600 mg/kg CPD (*P* < 0.05); however, this reduction was within the normal laboratory reference values. No significant changes were observed in other biochemical parameters compared with the control group (*P* > 0.05). Moreover, no changes in the relative weights or pathological changes in the heart, liver, spleen, lungs, or kidneys of mice were observed in our sub-chronic toxicity study. Because CPD is an edible plant and the effect of CPD on the gut microbiota was subsequently observed, we also examined the mice for lesions in the colon. The histomorphology of the mouse colon was normal in all groups.

Gut microbiota is an important part of the human body, and considerable research has demonstrated the critical impact of gut microbiota and its derivatives on human metabolism, immunity, and health [24, 40–42]. Age, antibiotic use, health status, and lifestyle can affect the structure and composition of the gut microbiota [43], and diet is one of the most important factors shaping the gut microbiota [44]. Many studies have reported that natural plants can interact with the gut microbiota [45, 46]. Therefore, we observed the changes of gut microbiota in mice after CPD intervention in the sub-chronic toxicity study to provide further evidence for its application. In the current study, intervention with CPD for 4 and 12 weeks exerted no effect on the alpha and beta diversities of the gut microbiota of mice in comparison with the control group (*P* > 0.05). The F/B ratio reflects gut microbiota dysbiosis and may change in obesity and other disease states [47], so the F/B ratio for each group was calculated. Similarly, compared to the control group, the different doses of CPD did not affect the ratio of F/B (*P* > 0.05). These results indicated that CPD had no effect on the structure and composition of the gut microbiota in mice.

Specific bacterial abundance changes were observed following CPD intervention. Exposure to 400 mg/kg CPD for 4 weeks significantly increased the abundance of *Rikenellaceae*, whereas 600 mg/kg CPD increased the abundance of *Alloprevotella* and *Prevotellaceae* by the Lefse analysis. When the intervention time was prolonged to 12 weeks, 600 mg/kg CPD enhanced the abundance of *Lachnospiraceae_NK4A136_group* significantly. Studies have shown that *Rikenellaceae* is involved in inflammatory responses and negatively correlates with the expression of pro-inflammatory cytokines [48]. In addition, *Rikenellaceae* is associated with metabolic diseases [49] and has also been shown to reduce adipogenesis [50]. *Alloprevotella* can produce short-chain fatty acids (SCFA) and protect the intestinal barrier [51]. Studies have shown that glycoursodeoxycholic acid attenuates the development of atherosclerosis, accompanied by an increased abundance of *Alloprevotella* [52]. CPD intervention enhanced the abundance of *Rikenellaceae* and *Alloprevotella*, suggesting a possible protective effect of CPD on metabolism. *Prevotellaceae* is sensitive to dietary fiber, and the improvement in cognition and glucose metabolism caused by dietary fiber is often accompanied by an increase in the abundance of *Prevotellaceae* [53, 54]. Consistent with other studies, our study similarly found that intervention with CPD increased the abundance of *Prevotellaceae*. CPD at 600 mg/kg can enrich *Lachnospiraceae_NK4A136_group* after an intervention of 12 weeks, which can produce short-chain fatty acids involved in the alleviation of obesity status and insulin resistance [55]. These results indicate that supplementation with CPD may result in an elevated abundance of beneficial gut bacteria, which may have a positive effect on health.

In this study, the oral toxicity of CPD in mice was examined by both acute and sub-chronic toxicity trials. In order to investigate its suitability in routine applications and provide a basis for its application as a nutraceutical, we focused on evaluating the toxicity of CPD rather than the identification of the active components. In addition to the pharmacological effects of CPD, which should be explored in subsequent studies, the active components of CPD should also be investigated. The results of the present study demonstrated the low toxicity of CPD. CPD intervention enhanced the abundance of several beneficial gut bacteria without disturbing the normal gut microbiota structure in mice. Conclusively, the study findings support the potential application of CPD as a functional food and natural herbal medicine.

## Data Availability

Sequencing raw data of the gut microbiota was uploaded to NCBI SRA database (BioProject ID: PRJNA976252).

## Abbreviations

ALT: Alanine aminotransferase
AST: Aspartate aminotransferase
ASVs: Amplicon Sequence Variants
CPD: *Cucurbita pepo cv Dayangua*
CHO: Cholesterol
CK: Creatine kinase
CREA: Creatine
Gran#: Granulocyte count
HGB: Hemoglobin
HCT: Hematocrit
HDL: High-density lipoprotein
LD50: Median lethal dose
Lymph#: Lymphocyte count
LDL: Low-density lipoprotein
LDH: Lactate dehydrogenase
Lefse: Linear discriminant analysis effect size
MCHC: Mean corpuscular hemoglobin concentration
Mon#: Monocyte count
OECD: Organization for Economic Cooperation and Development
PLT: Platelet count
PCoA: Principal Co-ordinates Analysis
RBC: Red blood cell count
RDW: Red blood cell distribution width
TG: Triglycerides
UDP: Up-and-Down-Procedure
UREA: Urea
WBC: White blood cell count
